# Supplementary figures and images for: Simultaneous SNP selection and adjustment for population structure in high dimensional prediction models
Source: PLoS Genet. 2020 May 4;16(5):e1008766. doi: 10.1371/journal.pgen.1008766 (PMC7224575; doi:10.1371/journal.pgen.1008766)

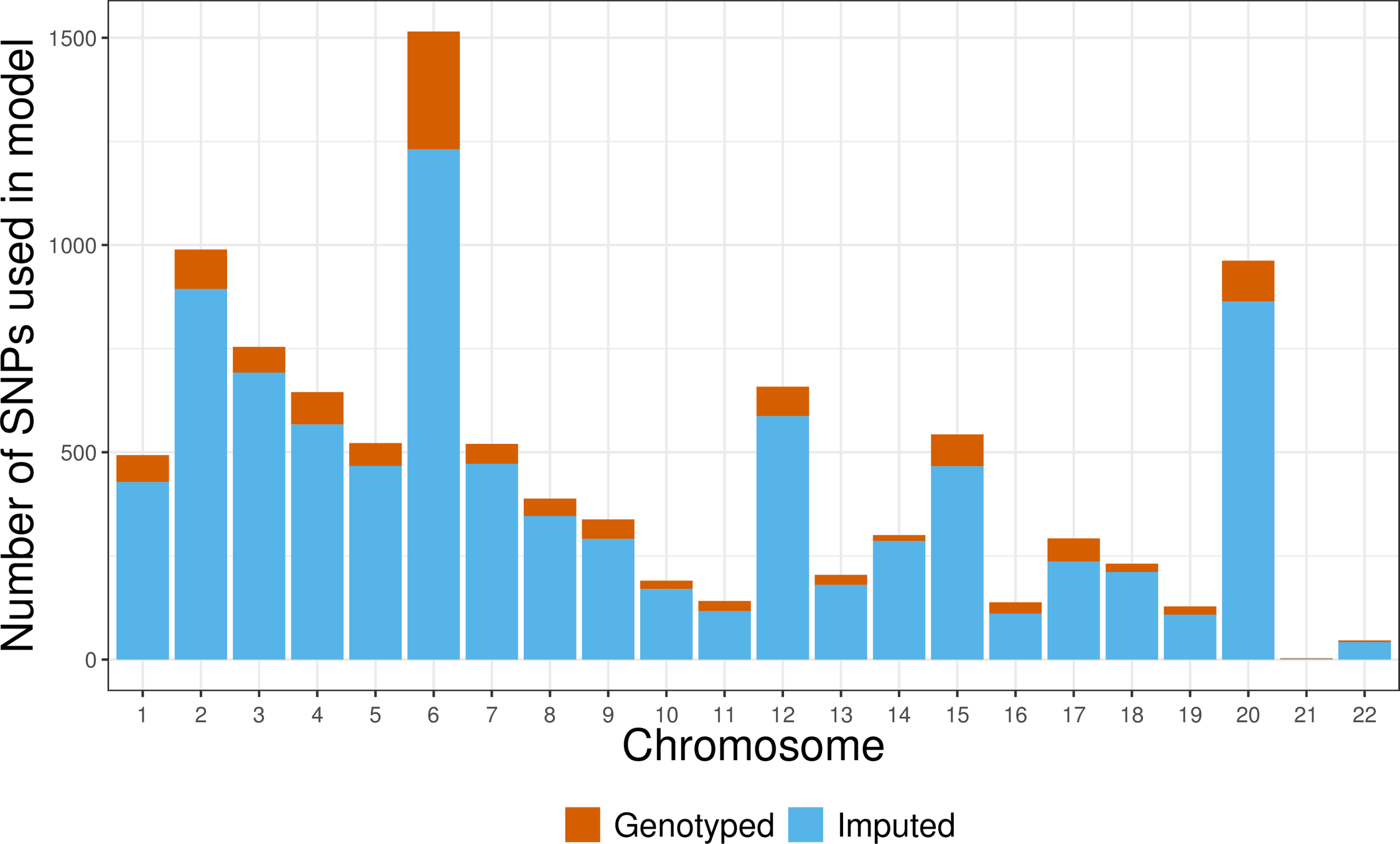

Supplement: S1 Fig — Distribution of SNPs used in UK Biobank analysis by chromosome and whether or not the SNP was imputed. (TIF) [file pgen.1008766.s001.tif]

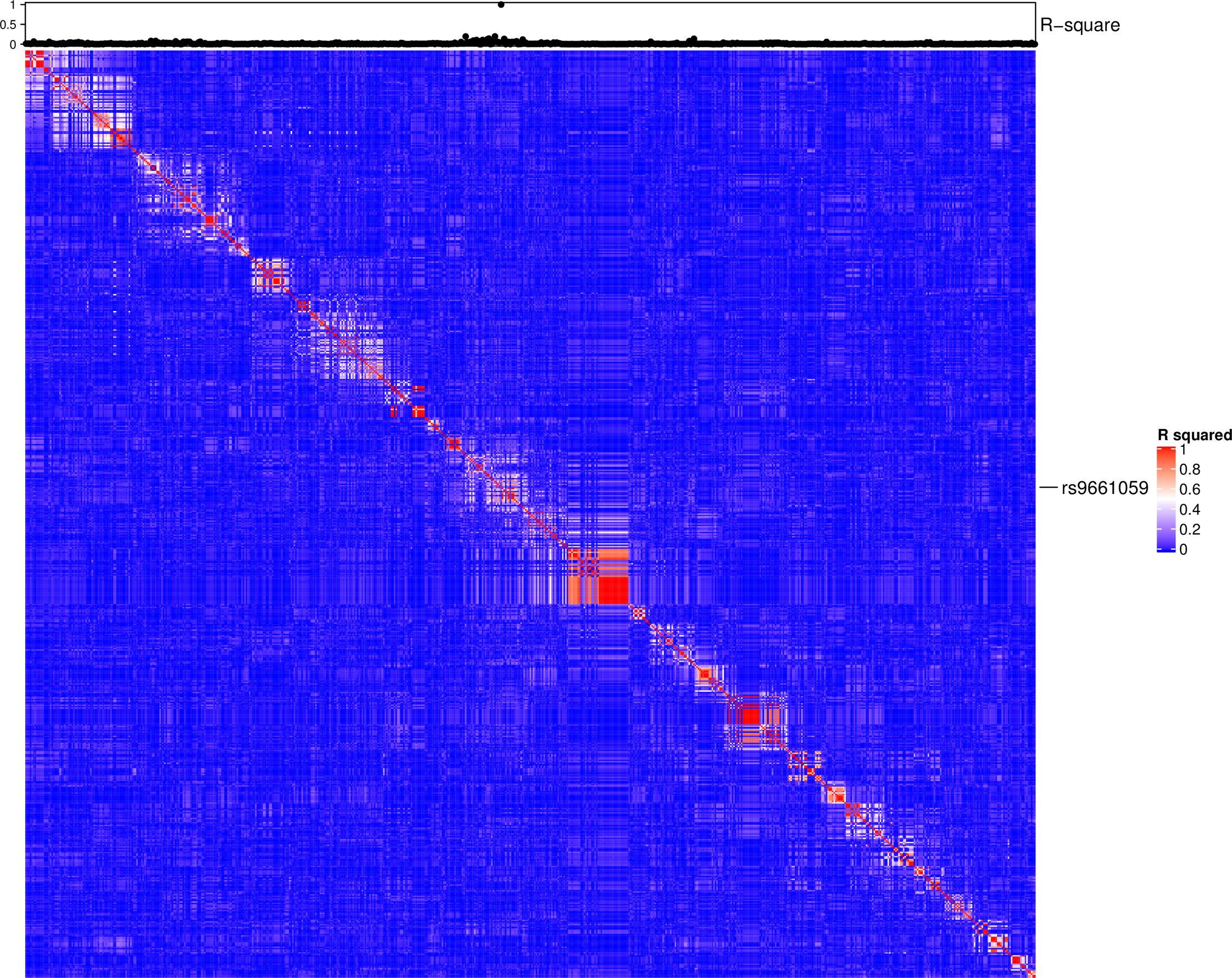

Supplement: S2 Fig — We illustrate the LD structure among the markers in the GAW20 dataset. We show the pairwise r2 for 655 SNPs within a 1Mb-window around the causal SNP rs9661059 (indicated) that we focused on. The dotplot above the heatmap denotes r2 between each SNP and the causal SNP. It is clear that although strong correlation does exist between some SNPs, none of these nearby SNPs is correlated with the causal SNP. The only dot denoting an r2 = 1 represents the causal SNP itself. (TIF) [file pgen.1008766.s002.tif]

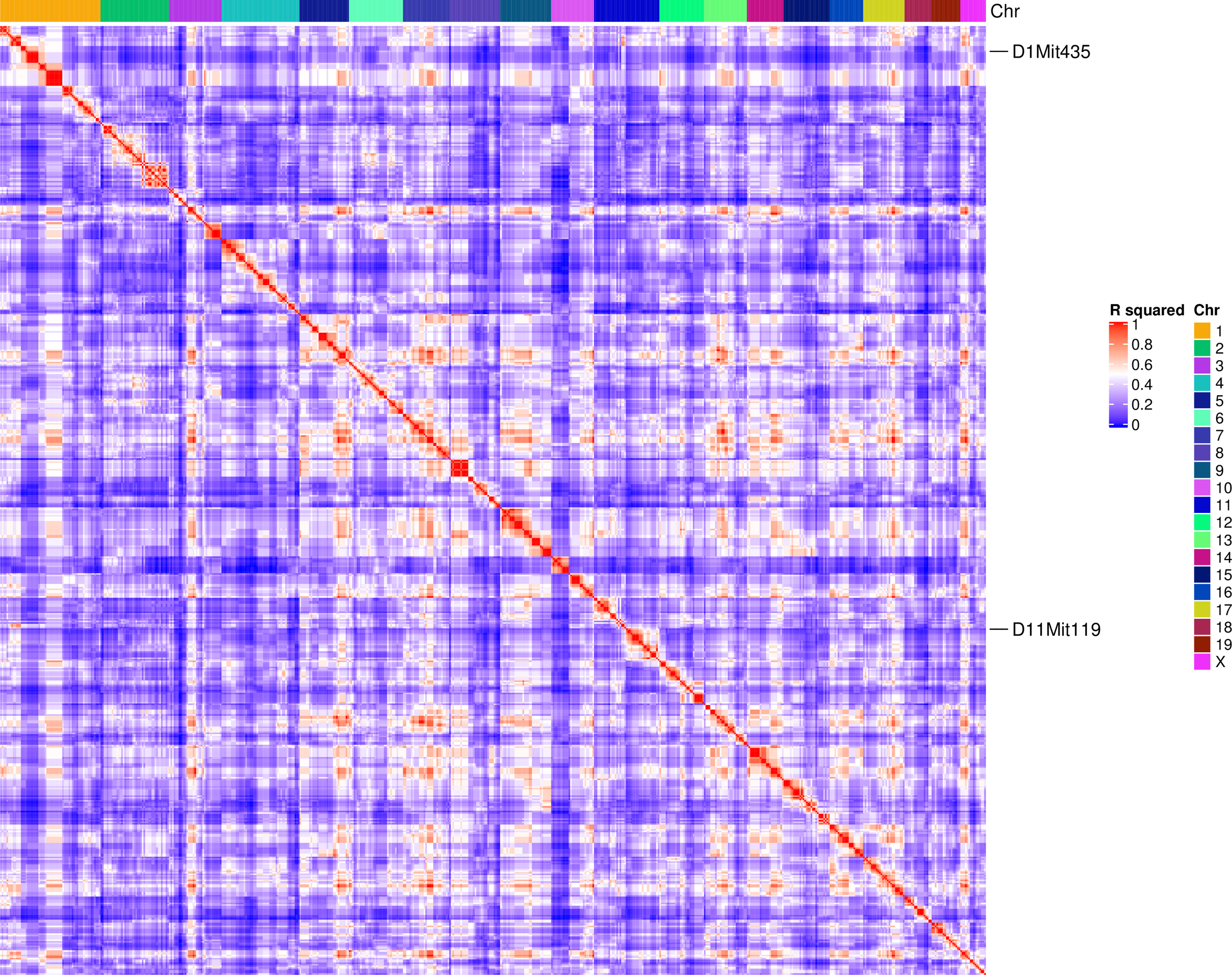

Supplement: S3 Fig — We illustrate the LD structure among the markers in the mouse dataset. Shown is the pairwise r2 for all microsatellite markers. It is clear that many markers are considerably strongly correlated with each other, as we expected. (TIF) [file pgen.1008766.s003.tif]
