# Supplementary material for: Simultaneous SNP selection and adjustment for population structure in high dimensional prediction models
Source: PLoS Genet. 2020 May 4;16(5):e1008766. doi: 10.1371/journal.pgen.1008766 (PMC7224575; doi:10.1371/journal.pgen.1008766)
Supplement: S1 Text — We provide a full derivation of the algorithm used to solve the ggmix objective function. (PDF) [file pgen.1008766.s004.pdf]

S1 Text

Block Coordinate Descent Algorithm

Model Set-up

Let $i = 1, \ldots, N$ be a grouping index, $j = 1, \ldots, n_i$ the observation index within a group and $N_T = \sum_{i=1}^{N} n_i$ the total number of observations. For each group let $\boldsymbol{y}_i = (y_1, \ldots, y_{n_i})$ be the observed vector of responses or phenotypes, $\mathbf{X}_i$ an $n_i \times (p+1)$ design matrix (with the column of 1s for the intercept), $\boldsymbol{b}_i$ a group-specific random effect vector of length n_i and $\boldsymbol{\varepsilon}_i = (\varepsilon_{i1}, \ldots, \varepsilon_{in_i})$ the individual error terms. Denote the stacked vectors $\mathbf{Y} = (\boldsymbol{y}_i, \ldots, \boldsymbol{y}_N)^T \in \mathbb{R}^{N_T \times 1}$, $\boldsymbol{b} = (\boldsymbol{b}_i, \ldots, \boldsymbol{b}_N)^T \in \mathbb{R}^{N_T \times 1}$, $\boldsymbol{\varepsilon} = (\boldsymbol{\varepsilon}_i, \ldots, \boldsymbol{\varepsilon}_N)^T \in \mathbb{R}^{N_T \times 1}$, and the stacked matrix $\mathbf{X} = (\mathbf{X}_1^T, \ldots, \mathbf{X}_N^T) \in \mathbb{R}^{N_T \times (p+1)}$. Furthermore, let $\boldsymbol{\beta} = (\beta_0, \beta_1, \ldots, \beta_p)^T \in \mathbb{R}^{(p+1) \times 1}$ be a vector of fixed effects regression coefficients corresponding to $\mathbf{X}$. We consider the following linear mixed model with a single random effect [1]:

$$\mathbf{Y} = \mathbf{X}\boldsymbol{\beta} + \boldsymbol{b} + \boldsymbol{\varepsilon} \tag{1}$$

where the random effect $\boldsymbol{b}$ and the error variance $\boldsymbol{\varepsilon}$ are assigned the distributions

$$\boldsymbol{b} \sim \mathcal{N}(0, \eta\sigma^2\boldsymbol{\Phi}) \qquad \boldsymbol{\varepsilon} \sim \mathcal{N}(0, (1-\eta)\sigma^2\mathbf{I}) \tag{2}$$

Here, $\boldsymbol{\Phi}_{N_T \times N_T}$ is a known positive semi-definite and symmetric covariance or kinship matrix calculated from SNPs sampled across the genome, $\mathbf{I}_{N_T \times N_T}$ is the identity matrix and parameters σ^2 and $\eta \in [0, 1]$ determine how the variance is divided between $\boldsymbol{b}$ and $\boldsymbol{\varepsilon}$. Note that η is also the narrow-sense heritability (h^2), defined as the proportion of phenotypic variance attributable to the additive genetic factors [2]. The joint density of $\mathbf{Y}$ is therefore

multivariate normal:

$$\mathbf{Y}|(\boldsymbol{\beta}, \eta, \sigma^2) \sim \mathcal{N}(\mathbf{X}\boldsymbol{\beta}, \eta\sigma^2\boldsymbol{\Phi} + (1-\eta)\sigma^2\mathbf{I}) \tag{3}$$

We consider the parameterization in (3) since maximization is easier over the compact set $\eta \in [0, 1]$ than over the unbounded interval $\delta \in [0, \infty)$ [1]. We define the complete parameter vector as $\boldsymbol{\Theta} := (\boldsymbol{\beta}, \eta, \sigma^2)$. The negative log-likelihood for (3) is given by

$$-\ell(\boldsymbol{\Theta}) \propto \frac{N_T}{2}\log(\sigma^2) + \frac{1}{2}\log\left(\det(\mathbf{V})\right) + \frac{1}{2\sigma^2}\left(\mathbf{Y} - \mathbf{X}\boldsymbol{\beta}\right)^T \mathbf{V}^{-1}\left(\mathbf{Y} - \mathbf{X}\boldsymbol{\beta}\right) \tag{4}$$

where $\mathbf{V} = \eta\boldsymbol{\Phi} + (1-\eta)\mathbf{I}$ and $\det(\mathbf{V})$ is the determinant of $\mathbf{V}$. Let $\boldsymbol{\Phi} = \mathbf{U}\mathbf{D}\mathbf{U}^T$ be the eigen (spectral) decomposition of the kinship matrix $\boldsymbol{\Phi}$, where $\mathbf{U}_{N_T \times N_T}$ is an orthonormal matrix of eigenvectors (i.e. $\mathbf{U}\mathbf{U}^T = \mathbf{I}$) and $\mathbf{D}_{N_T \times N_T}$ is a diagonal matrix of eigenvalues Λ_i. In the main text we show that $\mathbf{V}$ can then be further simplified to

$$\mathbf{V} = \mathbf{U}\widetilde{\mathbf{D}}\mathbf{U}^T \tag{5}$$

where

$$\widetilde{\mathbf{D}} = \text{diag}\left\{1 + \eta(\Lambda_1 - 1), 1 + \eta(\Lambda_2 - 1), \ldots, 1 + \eta(\Lambda_{N_T} - 1)\right\} \tag{6}$$

Since (6) is a diagonal matrix, its inverse is also a diagonal matrix:

$$\widetilde{\mathbf{D}}^{-1} = \text{diag}\left\{\frac{1}{1 + \eta(\Lambda_1 - 1)}, \frac{1}{1 + \eta(\Lambda_2 - 1)}, \ldots, \frac{1}{1 + \eta(\Lambda_{N_T} - 1)}\right\} \tag{7}$$

From (5) and (6), $\log(\det(\mathbf{V}))$ simplifies to

$$
\begin{aligned}
\log(\det(\mathbf{V})) &= \log\left(\det(\mathbf{U})\det\left(\widetilde{\mathbf{D}}\right)\det(\mathbf{U}^T)\right) \\
&= \log\left\{\prod_{i=1}^{N_T}(1+\eta(\Lambda_i-1))\right\} \\
&= \sum_{i=1}^{N_T}\log(1+\eta(\Lambda_i-1)) \qquad (8)
\end{aligned}
$$

since $\det(\mathbf{U}) = 1$. It also follows from (5) that

$$
\begin{aligned}
\mathbf{V}^{-1} &= \left(\mathbf{U}\widetilde{\mathbf{D}}\mathbf{U}^T\right)^{-1} \\
&= \left(\mathbf{U}^T\right)^{-1}\left(\widetilde{\mathbf{D}}\right)^{-1}\mathbf{U}^{-1} \\
&= \mathbf{U}\widetilde{\mathbf{D}}^{-1}\mathbf{U}^T \qquad (9)
\end{aligned}
$$

since for an orthonormal matrix $\mathbf{U}^{-1} = \mathbf{U}^T$. Substituting (7), (8) and (9) into (4) the negative log-likelihood becomes

$$
-\ell(\boldsymbol{\Theta}) = \frac{N_T}{2}\log(\sigma^2) + \frac{1}{2}\sum_{i=1}^{N_T}\log(1+\eta(\Lambda_i-1)) + \frac{1}{2\sigma^2}\sum_{i=1}^{N_T}\frac{\left(\widetilde{Y}_i - \sum_{j=0}^{p}\widetilde{X}_{ij+1}\beta_j\right)^2}{1+\eta(\Lambda_i-1)} \qquad (10)
$$

where $\widetilde{\mathbf{Y}} = \mathbf{U}^T\mathbf{Y}$, $\widetilde{\mathbf{X}} = \mathbf{U}^T\mathbf{X}$, $\widetilde{Y}_i$ denotes the ith element of $\widetilde{\mathbf{Y}}$, $\widetilde{X}_{ij}$ is the i, j^{th} entry of $\widetilde{\mathbf{X}}$ and $\mathbf{1}$ is a column vector of N_T ones.

Penalized Maximum Likelihood Estimator

We define the $p+3$ length vector of parameters $\boldsymbol{\Theta} := (\Theta_0, \Theta_1, \ldots, \Theta_{p+1}, \Theta_{p+2}, \Theta_{p+3}) = (\boldsymbol{\beta}, \eta, \sigma^2)$ where $\boldsymbol{\beta} \in \mathbb{R}^{p+1}, \eta \in [0,1], \sigma^2 > 0$. In what follows, $p+2$ and $p+3$ are the indices in $\boldsymbol{\Theta}$ for η and σ^2, respectively. In light of our goals to select variables associated with the response in high-dimensional data, we propose to place a constraint on the magnitude of the regression coefficients. This can be achieved by adding a penalty term to the likelihood

function (10). The penalty term is a necessary constraint because in our applications, the sample size is much smaller than the number of predictors. We define the following objective function:

$$Q_\lambda(\boldsymbol{\Theta}) = f(\boldsymbol{\Theta}) + \lambda \sum_{j \neq 0} v_j P_j(\beta_j) \tag{11}$$

where $f(\boldsymbol{\Theta}) := -\ell(\boldsymbol{\Theta})$ is defined in (10), $P_j(\cdot)$ is a penalty term on the fixed regression coefficients $\beta_1, \ldots, \beta_{p+1}$ (we do not penalize the intercept) controlled by the nonnegative regularization parameter λ, and v_j is the penalty factor for jth covariate. These penalty factors serve as a way of allowing parameters to be penalized differently. Note that we do not penalize η or σ^2. An estimate of the regression parameters $\widehat{\boldsymbol{\Theta}}_\lambda$ is obtained by

$$\widehat{\boldsymbol{\Theta}}_\lambda = \arg\min_{\boldsymbol{\Theta}} Q_\lambda(\boldsymbol{\Theta}) \tag{12}$$

We use a general purpose block coordinate descent algorithm (CGD) [3] to solve (12). At each iteration, the algorithm approximates the negative log-likelihood $f(\cdot)$ in $Q_\lambda(\cdot)$ by a strictly convex quadratic function and then applies block coordinate decent to generate a decent direction followed by an inexact line search along this direction [3]. For continuously differentiable $f(\cdot)$ and convex and block-separable $P(\cdot)$ (i.e. $P(\boldsymbol{\beta}) = \sum_i P_i(\beta_i)$), [3] show that the solution generated by the CGD method is a stationary point of $Q_\lambda(\cdot)$ if the coordinates are updated in a Gauss-Seidel manner i.e. $Q_\lambda(\cdot)$ is minimized with respect to one parameter while holding all others fixed. The CGD algorithm can thus be run in parallel and therefore suited for large p settings. It has been successfully applied in fixed effects models (e.g. [4], [5]) and [6] for mixed models with an ℓ_1 penalty. Following Tseng and Yun [3], the CGD algorithm is given by Algorithm 1.

Algorithm 1: Coordinate Gradient Descent Algorithm to solve (12)

Set the iteration counter $k \leftarrow 0$ and choose initial values for the parameter vector $\mathbf{\Theta}^{(0)}$;

repeat

Approximate the Hessian $\nabla^2 f(\mathbf{\Theta}^{(k)})$ by a symmetric matix $H^{(k)}$:

$$H^{(k)} = \text{diag}\left[\min\left\{\max\left\{\left[\nabla^2 f(\mathbf{\Theta}^{(k)})\right]_{jj}, c_{min}\right\} c_{max}\right\}\right]_{j=1,\ldots,p} \tag{13}$$

for $j = 1, \ldots, p$ **do**

Solve the descent direction $d^{(k)} := d_{H^{(k)}}(\Theta_j^{(k)})$;

if $\Theta_j^{(k)} \in \{\beta_1, \ldots, \beta_p\}$ **then**

$$d_{H^{(k)}}(\Theta_j^{(k)}) \leftarrow \arg\min_d \left\{\nabla f(\Theta_j^{(k)})d + \frac{1}{2}d^2 H_{jj}^{(k)} + \lambda P(\Theta_j^{(k)} + d)\right\} \tag{14}$$

end

end

Choose a stepsize;

$$\alpha_j^{(k)} \leftarrow \text{line search given by the Armijo rule}$$

Update;

$$\widehat{\Theta}_j^{(k+1)} \leftarrow \widehat{\Theta}_j^{(k)} + \alpha_j^{(k)} d^{(k)}$$

Update;

$$\widehat{\eta}^{(k+1)} \leftarrow \arg\min_\eta \frac{1}{2}\sum_{i=1}^{N_T} \log(1 + \eta(\Lambda_i - 1)) + \frac{1}{2\sigma^{2\,(k)}} \sum_{i=1}^{N_T} \frac{\left(\widetilde{Y}_i - \sum_{j=0}^p \widetilde{X}_{ij+1}\beta_j^{(k+1)}\right)^2}{1 + \eta(\Lambda_i - 1)} \tag{15}$$

Update;

$$\widehat{\sigma^2}^{\,(k+1)} \leftarrow \frac{1}{N_T}\sum_{i=1}^{N_T} \frac{\left(\widetilde{Y}_i - \sum_{j=0}^p \widetilde{X}_{ij+1}\beta_j^{(k+1)}\right)^2}{1 + \eta^{(k+1)}(\Lambda_i - 1)} \tag{16}$$

$k \leftarrow k + 1$

until *convergence criterion is satisfied*;

The Armijo rule is defined as follows [3]:

Choose $\alpha_{init}^{(k)} > 0$ and let $\alpha^{(k)}$ be the largest element of $\left\{\alpha_{init}^{k}\delta^{r}\right\}_{r=0,1,2,\ldots}$ satisfying

$$Q_\lambda(\Theta_j^{(k)} + \alpha^{(k)} d^{(k)}) \leq Q_\lambda(\Theta_j^{(k)}) + \alpha^{(k)} \varrho \Delta^{(k)} \tag{17}$$

where $0 < \delta < 1$, $0 < \varrho < 1$, $0 \leq \gamma < 1$ and

$$\Delta^{(k)} := \nabla f(\Theta_j^{(k)}) d^{(k)} + \gamma (d^{(k)})^2 H_{jj}^{(k)} + \lambda P(\Theta_j^{(k)} + d^{(k)}) - \lambda P(\Theta^{(k)}) \tag{18}$$

Common choices for the constants are $\delta = 0.1$, $\varrho = 0.001$, $\gamma = 0$, $\alpha_{init}^{(k)} = 1$ for all k [6].

Below we detail the specifics of Algorithm 1 for the ℓ_1 penalty.

ℓ_1 penalty

The objective function is given by

$$Q_\lambda(\boldsymbol{\Theta}) = f(\boldsymbol{\Theta}) + \lambda |\boldsymbol{\beta}| \tag{19}$$

Descent Direction

For simplicity, we remove the iteration counter (k) from the derivation below.

For $\Theta_j^{(k)} \in \{\beta_1, \ldots, \beta_p\}$, let

$$d_H(\Theta_j) = \arg\min_d G(d) \tag{20}$$

where

$$G(d) = \nabla f(\Theta_j) d + \frac{1}{2} d^2 H_{jj} + \lambda |\Theta_j + d|$$

Since $G(d)$ is not differentiable at $-\Theta_j$, we calculate the subdifferential $\partial G(d)$ and search for d with $0 \in \partial G(d)$:

$$\partial G(d) = \nabla f(\Theta_j) + d H_{jj} + \lambda u \tag{21}$$

where

$$u = \begin{cases} 1 & \text{if} \quad d > -\Theta_j \\ -1 & \text{if} \quad d < -\Theta_j \\ [-1, 1] & \text{if} \quad d = \Theta_j \end{cases} \tag{22}$$

We consider each of the three cases in (21) below

1. $d > -\Theta_j$

$$\partial G(d) = \nabla f(\Theta_j) + dH_{jj} + \lambda = 0$$
$$d = \frac{-(\nabla f(\Theta_j) + \lambda)}{H_{jj}}$$

Since $\lambda > 0$ and $H_{jj} > 0$, we have

$$\frac{-(\nabla f(\Theta_j) - \lambda)}{H_{jj}} > \frac{-(\nabla f(\Theta_j) + \lambda)}{H_{jj}} = d \overset{\text{def}}{>} -\Theta_j$$

The solution can be written compactly as

$$d = \text{mid}\left\{\frac{-(\nabla f(\Theta_j) - \lambda)}{H_{jj}}, -\Theta_j, \frac{-(\nabla f(\Theta_j) + \lambda)}{H_{jj}}\right\}$$

where mid $\{a, b, c\}$ denotes the median (mid-point) of a, b, c [3].

2. $d < -\Theta_j$

$$\partial G(d) = \nabla f(\Theta_j) + dH_{jj} - \lambda = 0$$
$$d = \frac{-(\nabla f(\Theta_j) - \lambda)}{H_{jj}}$$

Since $\lambda > 0$ and $H_{jj} > 0$, we have

$$\frac{-(\nabla f(\Theta_j) + \lambda)}{H_{jj}} < \frac{-(\nabla f(\Theta_j) - \lambda)}{H_{jj}} = d \overset{\text{def}}{<} -\Theta_j$$

Again, the solution can be written compactly as

$$d = \text{mid}\left\{\frac{-(\nabla f(\Theta_j) - \lambda)}{H_{jj}}, -\Theta_j, \frac{-(\nabla f(\Theta_j) + \lambda)}{H_{jj}}\right\}$$

3. $d_j = -\Theta_j$

   There exists $u \in [-1, 1]$ such that

$$\begin{aligned} \partial G(d) &= \nabla f(\Theta_j) + dH_{jj} + \lambda u = 0 \\ d &= \frac{-(\nabla f(\Theta_j) + \lambda u)}{H_{jj}} \end{aligned}$$

   For $-1 \leq u \leq 1$, $\lambda > 0$ and $H_{jj} > 0$ we have

$$\frac{-(\nabla f(\Theta_j) + \lambda)}{H_{jj}} \leq d \stackrel{\text{def}}{=} -\Theta_j \leq \frac{-(\nabla f(\Theta_j) - \lambda)}{H_{jj}}$$

   The solution can again be written compactly as

$$d = \text{mid}\left\{\frac{-(\nabla f(\Theta_j) - \lambda)}{H_{jj}}, -\Theta_j, \frac{-(\nabla f(\Theta_j) + \lambda)}{H_{jj}}\right\}$$

We see all three cases lead to the same solution for (20). Therefore the descent direction for $\Theta_j^{(k)} \in \{\beta_1, \ldots, \beta_p\}$ for the ℓ_1 penalty is given by

$$d = \text{mid}\left\{\frac{-(\nabla f(\beta_j) - \lambda)}{H_{jj}}, -\beta_j, \frac{-(\nabla f(\beta_j) + \lambda)}{H_{jj}}\right\} \tag{23}$$

Solution for the β parameter

If the Hessian $\nabla^2 f(\boldsymbol{\Theta}^{(k)}) > 0$ then $H^{(k)}$ defined in (13) is equal to $\nabla^2 f(\boldsymbol{\Theta}^{(k)})$. Using $\alpha_{init} = 1$, the largest element of $\left\{\alpha_{init}^{(k)} \delta^r\right\}_{r=0,1,2,\ldots}$ satisfying the Armijo Rule inequality is reached for

$\alpha^{(k)} = \alpha_{init}^{(k)} \delta^0 = 1$. The Armijo rule update for the $\boldsymbol{\beta}$ parameter is then given by

$$\beta_j^{(k+1)} \leftarrow \beta_j^{(k)} + d^{(k)}, \qquad j = 1, \ldots, p \tag{24}$$

Substituting the descent direction given by (23) into (24) we get

$$\beta_j^{(k+1)} = \text{mid}\left\{ \beta_j^{(k)} + \frac{-(\nabla f(\beta_j^{(k)}) - \lambda)}{H_{jj}}, 0, \beta_j^{(k)} + \frac{-(\nabla f(\beta_j^{(k)}) + \lambda)}{H_{jj}} \right\} \tag{25}$$

We can further simplify this expression. Let

$$w_i := \frac{1}{\sigma^2 \left(1 + \eta(\Lambda_i - 1)\right)} \tag{26}$$

.

Re-write the part depending on $\boldsymbol{\beta}$ of the negative log-likelihood in (10) as

$$g(\boldsymbol{\beta}^{(k)}) = \frac{1}{2} \sum_{i=1}^{N_T} w_i \left(\widetilde{Y}_i - \sum_{\ell \neq j} \widetilde{X}_{i\ell} \beta_\ell^{(k)} - \widetilde{X}_{ij} \beta_j^{(k)} \right)^2 \tag{27}$$

The gradient and Hessian are given by

$$\nabla f(\beta_j^{(k)}) := \frac{\partial}{\partial \beta_j^{(k)}} g(\boldsymbol{\beta}^{(k)}) = -\sum_{i=1}^{N_T} w_i \widetilde{X}_{ij} \left(\widetilde{Y}_i - \sum_{\ell \neq j} \widetilde{X}_{i\ell} \beta_\ell^{(k)} - \widetilde{X}_{ij} \beta_j^{(k)} \right) \tag{28}$$

$$H_{jj} := \frac{\partial^2}{\partial {\beta_j^{(k)}}^2} g(\boldsymbol{\beta}^{(k)}) = \sum_{i=1}^{N_T} w_i \widetilde{X}_{ij}^2 \tag{29}$$

Substituting (28) and (29) into $\beta_j^{(k)} + \frac{-(\nabla f(\beta_j^{(k)})-\lambda)}{H_{jj}}$

$$\begin{aligned}
&\beta_j^{(k)} + \frac{\sum_{i=1}^{N_T} w_i \widetilde{X}_{ij}\left(\widetilde{Y}_i - \sum_{\ell \neq j} \widetilde{X}_{i\ell}\beta_\ell^{(k)} - \widetilde{X}_{ij}\beta_j^{(k)}\right) + \lambda}{\sum_{i=1}^{N_T} w_i \widetilde{X}_{ij}^2} \\
&= \beta_j^{(k)} + \frac{\sum_{i=1}^{N_T} w_i \widetilde{X}_{ij}\left(\widetilde{Y}_i - \sum_{\ell \neq j} \widetilde{X}_{i\ell}\beta_\ell^{(k)}\right) + \lambda}{\sum_{i=1}^{N_T} w_i \widetilde{X}_{ij}^2} - \frac{\sum_{i=1}^{N_T} w_i \widetilde{X}_{ij}^2 \beta_j^{(k)}}{\sum_{i=1}^{N_T} w_i \widetilde{X}_{ij}^2} \\
&= \frac{\sum_{i=1}^{N_T} w_i \widetilde{X}_{ij}\left(\widetilde{Y}_i - \sum_{\ell \neq j} \widetilde{X}_{i\ell}\beta_\ell^{(k)}\right) + \lambda}{\sum_{i=1}^{N_T} w_i \widetilde{X}_{ij}^2} \qquad (30)
\end{aligned}$$

Similarly, substituting (28) and (29) in $\beta_j^{(k)} + \frac{-(\nabla f(\beta_j^{(k)})+\lambda)}{H_{jj}}$ we get

$$\frac{\sum_{i=1}^{N_T} w_i \widetilde{X}_{ij}\left(\widetilde{Y}_i - \sum_{\ell \neq j} \widetilde{X}_{i\ell}\beta_\ell^{(k)}\right) - \lambda}{\sum_{i=1}^{N_T} w_i \widetilde{X}_{ij}^2} \qquad (31)$$

Finally, substituting (30) and (31) into (25) we get

$$\begin{aligned}
\beta_j^{(k+1)} &= \text{mid}\left\{\frac{\sum_{i=1}^{N_T} w_i \widetilde{X}_{ij}\left(\widetilde{Y}_i - \sum_{\ell \neq j} \widetilde{X}_{i\ell}\beta_\ell^{(k)}\right) - \lambda}{\sum_{i=1}^{N_T} w_i \widetilde{X}_{ij}^2}, 0, \frac{\sum_{i=1}^{N_T} w_i \widetilde{X}_{ij}\left(\widetilde{Y}_i - \sum_{\ell \neq j} \widetilde{X}_{i\ell}\beta_\ell^{(k)}\right) + \lambda}{\sum_{i=1}^{N_T} w_i \widetilde{X}_{ij}^2}\right\} \\
&= \frac{\mathcal{S}_\lambda\left(\sum_{i=1}^{N_T} w_i \widetilde{X}_{ij}\left(\widetilde{Y}_i - \sum_{\ell \neq j} \widetilde{X}_{i\ell}\beta_\ell^{(k)}\right)\right)}{\sum_{i=1}^{N_T} w_i \widetilde{X}_{ij}^2} \qquad (32)
\end{aligned}$$

Where $\mathcal{S}_\lambda(x)$ is the soft-thresholding operator

$$\mathcal{S}_\lambda(x) = \text{sign}(x)(|x| - \lambda)_+$$

sign(x) is the signum function

$$\text{sign}(x) = \begin{cases} -1 & x < 0 \\ 0 & x = 0 \\ 1 & x > 0 \end{cases}$$

and $(x)_+ = \max(x, 0)$.

References

[1] Pirinen M, Donnelly P, Spencer CC, et al. Efficient computation with a linear mixed model on large-scale data sets with applications to genetic studies. The Annals of Applied Statistics. 2013;7(1):369–390. 1, 2

[2] Manolio TA, Collins FS, Cox NJ, Goldstein DB, Hindorff LA, Hunter DJ, et al. Finding the missing heritability of complex diseases. Nature. 2009;461(7265):747. 1

[3] Tseng P, Yun S. A coordinate gradient descent method for nonsmooth separable minimization. Mathematical Programming. 2009;117(1):387–423. 4, 6, 7

[4] Meier L, Van De Geer S, Bühlmann P. The group lasso for logistic regression. Journal of the Royal Statistical Society: Series B (Statistical Methodology). 2008;70(1):53–71. 4

[5] Friedman J, Hastie T, Tibshirani R. Regularization paths for generalized linear models via coordinate descent. Journal of statistical software. 2010;33(1):1. 4

[6] Schelldorfer J, Bühlmann P, DE G, VAN S. Estimation for High-Dimensional Linear Mixed-Effects Models Using L1-Penalization. Scandinavian Journal of Statistics. 2011;38(2):197–214. 4, 6
