# Supplementary material for: Simultaneous SNP selection and adjustment for population structure in high dimensional prediction models
Source: PLoS Genet. 2020 May 4;16(5):e1008766. doi: 10.1371/journal.pgen.1008766 (PMC7224575; doi:10.1371/journal.pgen.1008766)
Supplement: S2 Text — We introduce the freely available and open source ggmix package in R available on CRAN (https://cran.r-project.org/package=ggmix). (PDF) [file pgen.1008766.s005.pdf]

S2 Text

ggmix Package Showcase

In this section we briefly introduce the freely available and open source ggmix package in R. More comprehensive documentation is available at https://sahirbhatnagar.com/ggmix. Note that this entire section is reproducible; the code and text are combined in an .Rnw[1] file and compiled using knitr [1].

Installation

The package can be installed from GitHub via

```
install.packages("pacman")
pacman::p_load_gh('sahirbhatnagar/ggmix')
```

To showcase the main functions in ggmix, we will use the simulated data which ships with the package and can be loaded via:

```
## library(ggmix)
data("admixed")
names(admixed)

##  [1] "ytrain"          "ytune"           "ytest"           "xtrain"
##  [5] "xtune"           "xtest"           "xtrain_lasso"    "xtune_lasso"
##  [9] "xtest_lasso"     "Xkinship"        "kin_train"       "kin_tune_train"
## [13] "kin_test_train"  "mu_train"        "causal"          "beta"
## [17] "not_causal"      "kinship"         "coancestry"      "PC"
## [21] "subpops"
```

For details on how this data was simulated, see help(admixed).

There are three basic inputs that ggmix needs:

1. Y: a continuous response variable
2. X: a matrix of covariates of dimension $N \times p$ where N is the sample size and p is the

[1]scripts available at https://github.com/sahirbhatnagar/ggmix/tree/pgen/manuscript

number of covariates

3. $\mathbf{\Phi}$: a kinship matrix

We can visualize the kinship matrix in the `admixed` data using the `popkin` package:

```
# need to install the package if you don't have it
# pacman::p_load_gh('StoreyLab/popkin')
popkin::plot_popkin(admixed$kin_train)
```

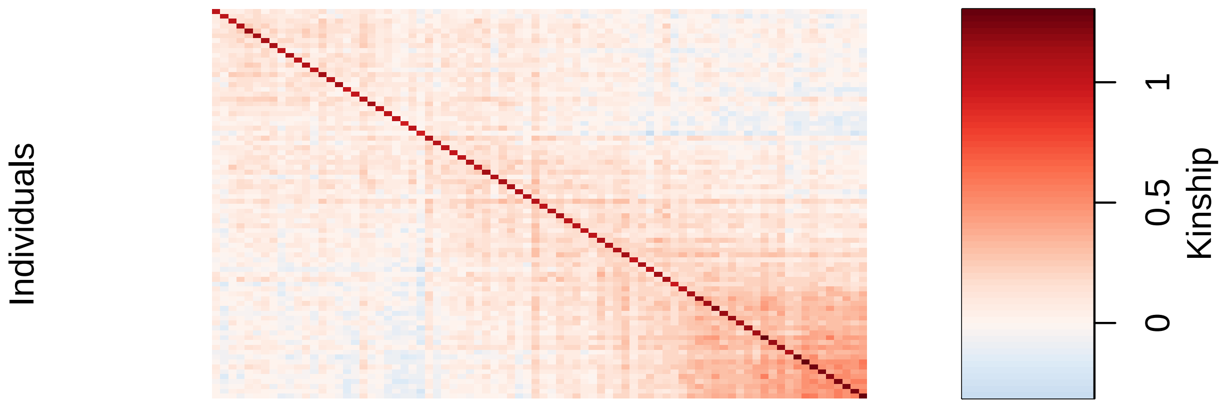

Fit the linear mixed model with Lasso Penalty

We will use the most basic call to the main function of this package, which is called `ggmix`. This function will by default fit a L_1 penalized linear mixed model (LMM) for 100 distinct values of the tuning parameter λ. It will choose its own sequence:

```
fit <- ggmix(x = admixed$xtrain,
                 y = admixed$ytrain,
                         kinship = admixed$kin_train)
names(fit)

##  [1] "result"       "ggmix_object" "n_design"     "p_design"     "lambda"
```

```
##  [6] "coef"         "b0"           "beta"         "df"           "eta"
## [11] "sigma2"       "nlambda"      "cov_names"    "call"

class(fit)

## [1] "lassofullrank" "ggmix_fit"
```

We can see the solution path for each variable by calling the `plot` method for objects of class `ggmix_fit`:

```
plot(fit)
```

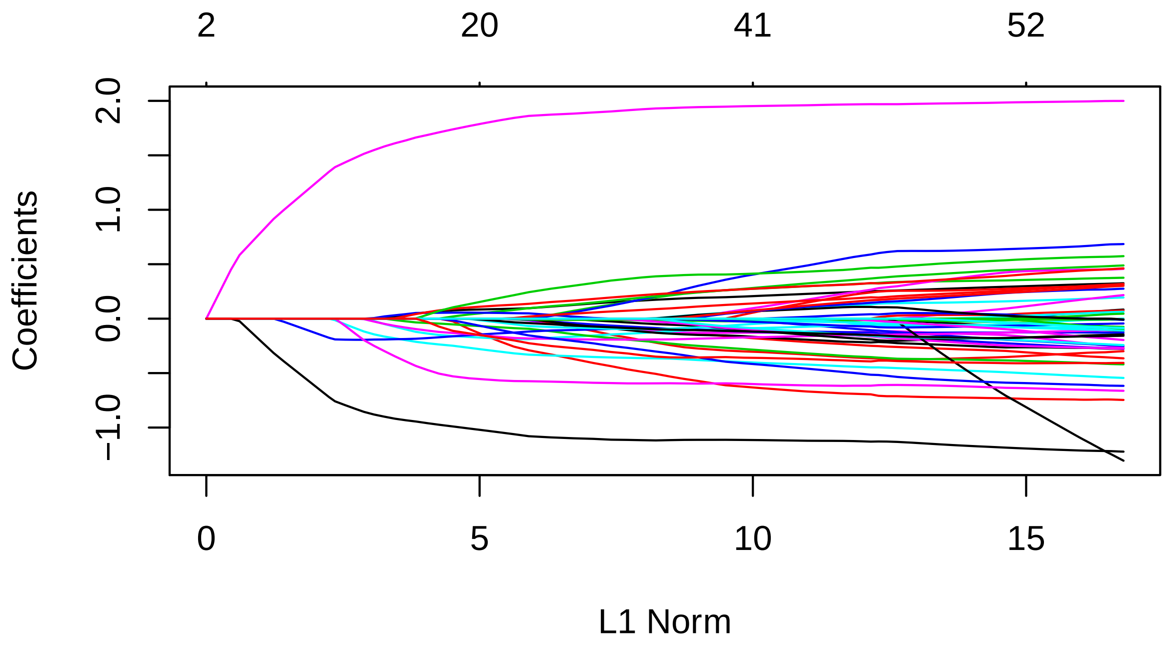

We can also get the coefficients for given value(s) of lambda using the `coef` method for objects of class `ggmix_fit`:

```
# only the first 5 coefficients printed here for brevity
coef(fit, s = c(0.1,0.02))[1:5, ]

## 5 x 2 Matrix of class "dgeMatrix"
##                       1            2
## (Intercept) -0.03715135  0.247105426
## X23          0.00000000  0.098030248
## X36          0.00000000 -0.013022250
## X38          0.00000000  0.005378361
```

```
## X40         0.00000000  0.004028934
```

Here, `s` specifies the value(s) of λ at which the extraction is made. The function uses linear interpolation to make predictions for values of `s` that do not coincide with the lambda sequence used in the fitting algorithm.

We can also get predictions ($X\widehat{\boldsymbol{\beta}}$) using the `predict` method for objects of class `ggmix_fit`:

```
# need to provide x to the predict function
# predict for the first 5 subjects
predict(fit, s = c(0.1,0.02), newx = admixed$xtest[1:5,])

##                1           2
## id26  2.30208546  2.45597763
## id39  0.87334032  1.62931898
## id45 -0.12296837 -0.06075786
## id52 -0.03715135 -0.97519671
## id53 -0.21046107 -0.23151040
```

Find the Optimal Value of the Tuning Parameter

We use the Generalized Information Criterion (GIC) to select the optimal value for λ. The default is $a_n = log(log(n)) * log(p)$ which corresponds to a high-dimensional BIC (HD-BIC):

```
# pass the fitted object from ggmix to the gic function:
hdbic <- gic(fit)
class(hdbic)

## [1] "ggmix_gic"     "lassofullrank" "ggmix_fit"

# we can also fit the BIC by specifying the an argument
bicfit <- gic(fit, an = log(length(admixed$ytrain)))
```

We can plot the HDBIC values against $\log(\lambda)$ using the `plot` method for objects of class `ggmix_gic`:

```r
plot(hdbic)
```

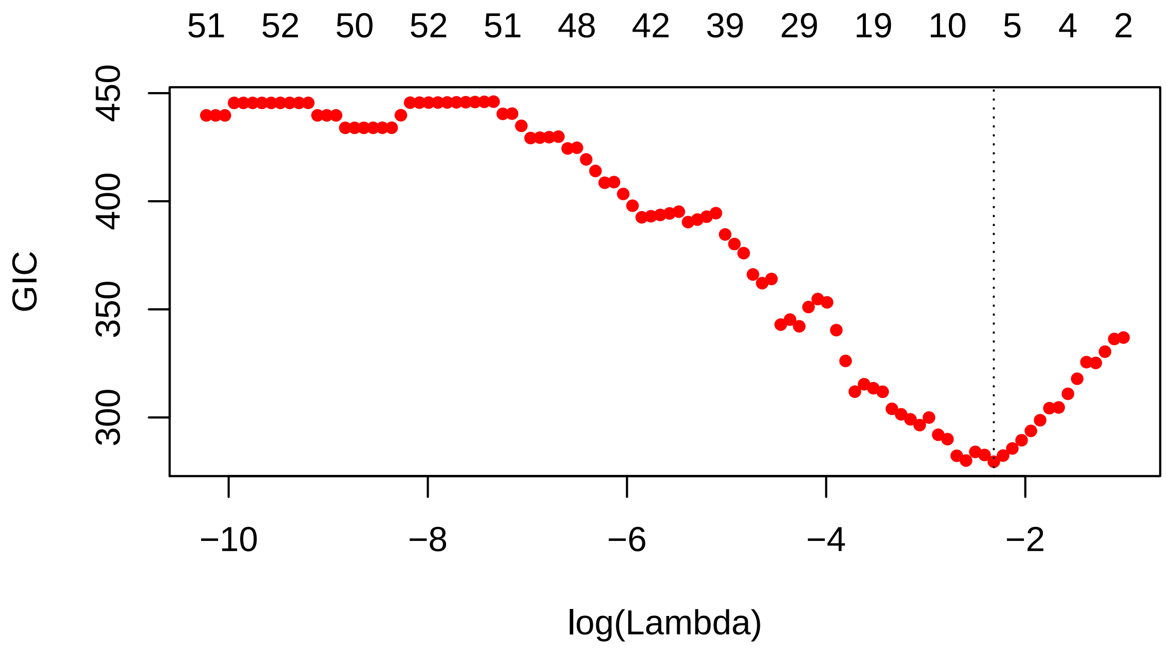

The optimal value for λ according to the HDBIC, i.e., the λ that leads to the minium HDBIC is:

```r
hdbic[["lambda.min"]]

## [1] 0.09862269
```

We can also plot the BIC results:

```r
plot(bicfit, ylab = "BIC")
```

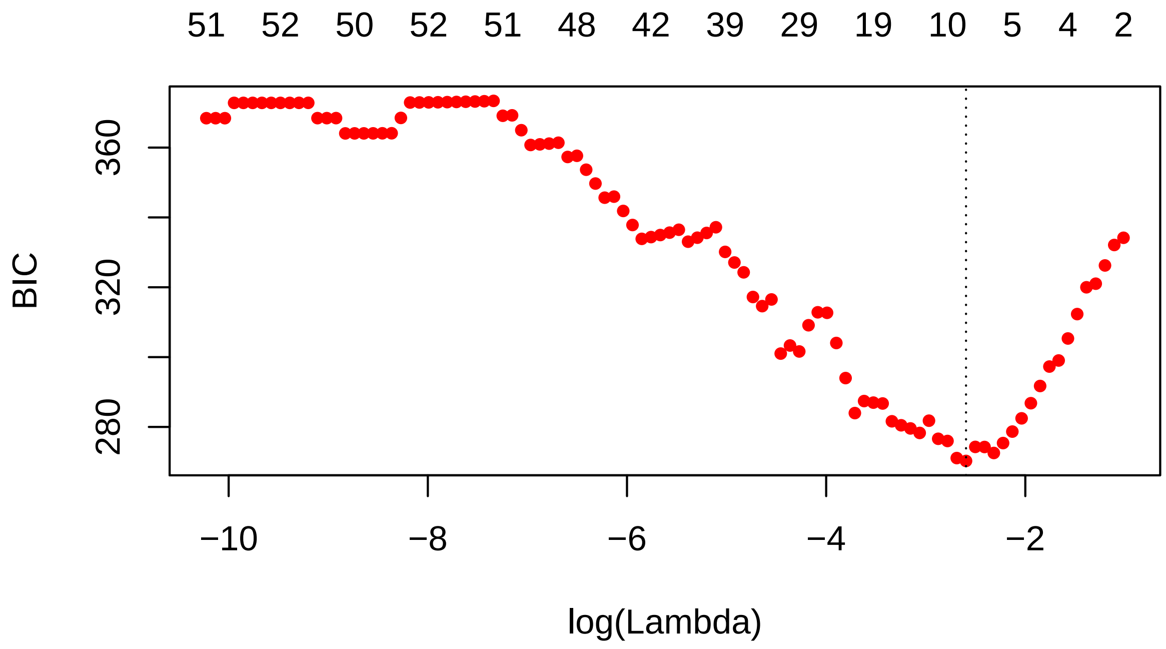


```
bicfit[["lambda.min"]]

## [1] 0.07460445
```

Get Coefficients Corresponding to Optimal Model

We can use the object outputted by the `gic` function to extract the coefficients corresponding to the selected model using the `coef` method for objects of class `ggmix_gic`:

```
coef(hdbic)[1:5, , drop = FALSE]

## 5 x 1 sparse Matrix of class "dgCMatrix"
##                       1
## (Intercept) -0.03660806
## X23         .
## X36         .
## X38         .
## X40         .
```

We can also extract just the nonzero coefficients which also provide the estimated variance components η and σ^2:

```r
coef(hdbic, type = "nonzero")
```

```
##                       1
## (Intercept) -0.03660806
## X302        -0.17607392
## X524         1.34951500
## X538        -0.72052613
## eta          0.99000000
## sigma2       1.60476289
```

We can also make predictions from the `hdbic` object, which by default will use the model corresponding to the optimal tuning parameter:

```r
predict(hdbic, newx = admixed$xtest[1:5,])
```

```
##                1
## id26  2.31027410
## id39  0.86922183
## id45 -0.12814532
## id52 -0.03660806
## id53 -0.21268198
```

Extracting Random Effects

The user can compute the random effects using the provided `ranef` method for objects of class `ggmix_gic`. This command will compute the estimated random effects for each subject using the parameters of the selected model:

```r
ranef(hdbic)[1:5]
```

```
## [1] -2.4889655  1.1834200 -0.5641832 -0.9310334 -0.3458703
```

Diagnostic Plots

We can also plot some standard diagnotic plots such as the observed vs. predicted response, QQ-plots of the residuals and random effects and the Tukey-Anscombe plot. These can be plotted using the `plot` method on a `ggmix_gic` object as shown below.

Observed vs. Predicted Response

```
plot(hdbic, type = "predicted", newx = admixed$xtrain, newy = admixed$ytrain)
```

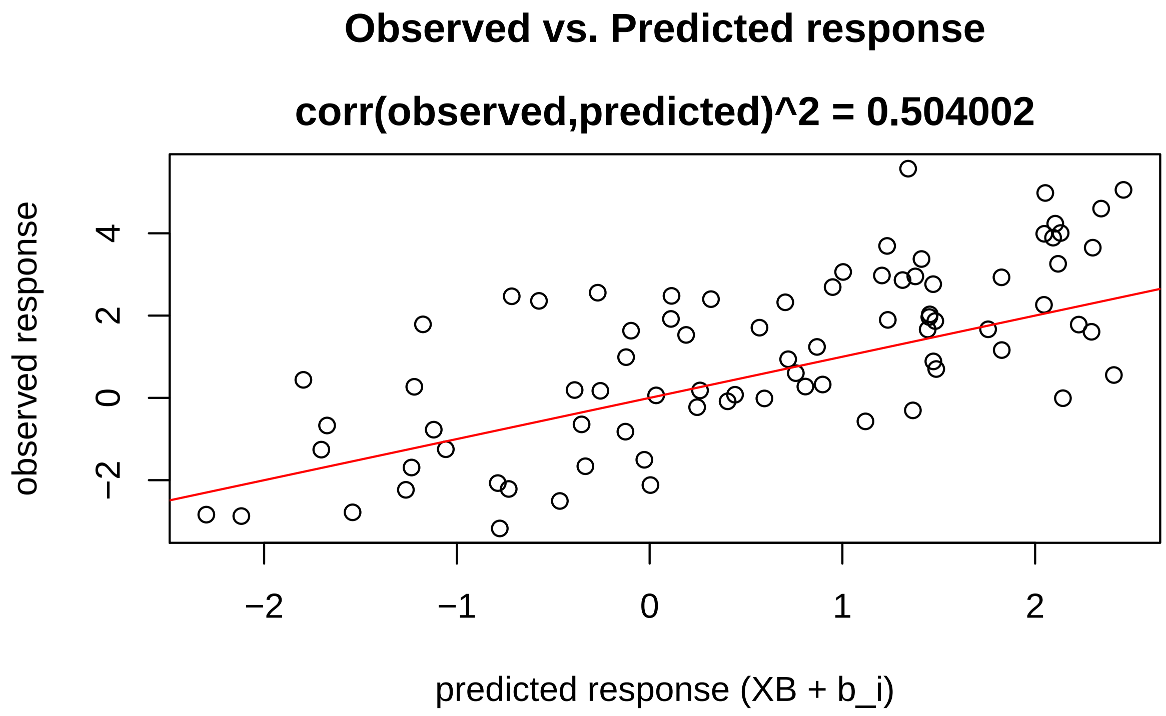

QQ-plots for Residuals and Random Effects

```
plot(hdbic, type = "QQranef", newx = admixed$xtrain, newy = admixed$ytrain)
```

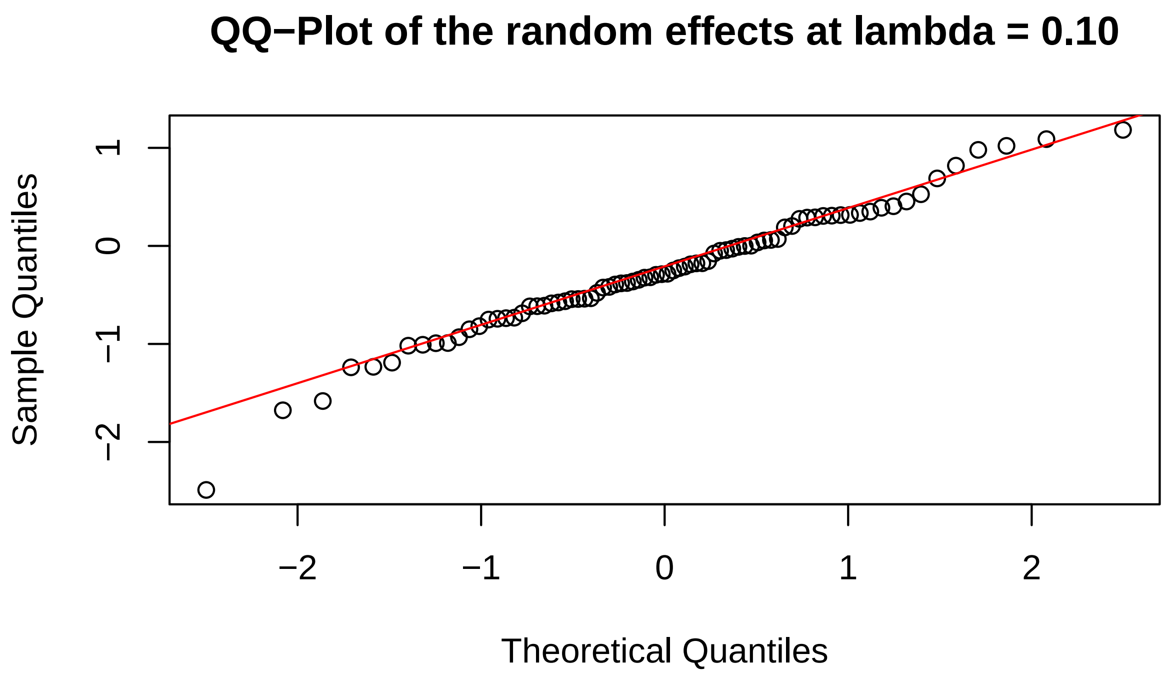


```r
plot(hdbic, type = "QQresid", newx = admixed$xtrain, newy = admixed$ytrain)
```

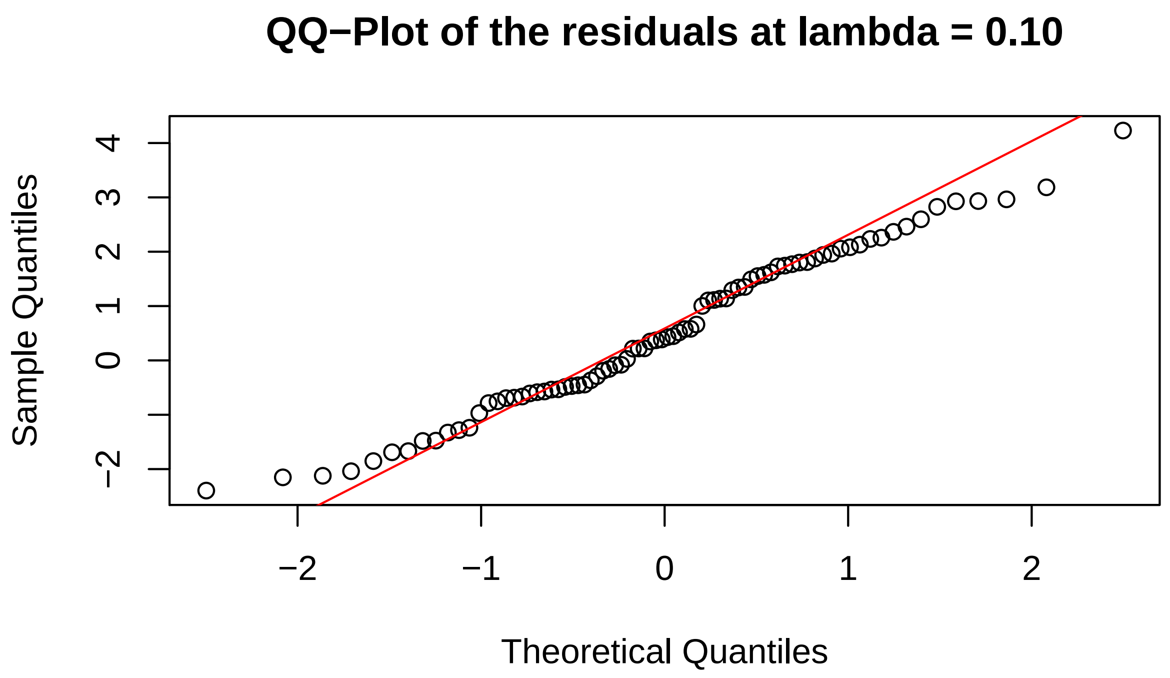

Tukey-Anscombe Plot

```
plot(hdbic, type = "Tukey", newx = admixed$xtrain, newy = admixed$ytrain)
```

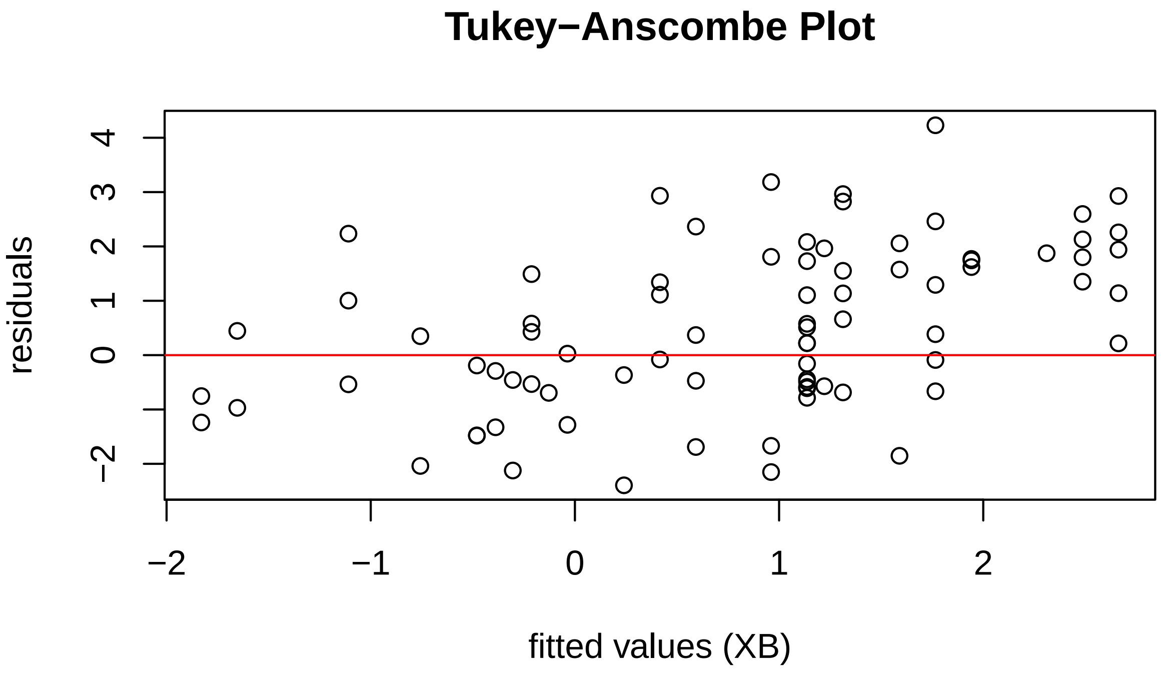

References

[1] Xie Y. Dynamic Documents with R and knitr. vol. 29. CRC Press; 2015. 1
