# Supplementary material for: Simultaneous SNP selection and adjustment for population structure in high dimensional prediction models
Source: PLoS Genet. 2020 May 4;16(5):e1008766. doi: 10.1371/journal.pgen.1008766 (PMC7224575; doi:10.1371/journal.pgen.1008766)
Supplement: Attachment — Submitted filename: response_to_reviewers.pdf [file pgen.1008766.s006.pdf]

December 8, 2019

Re: Simultaneous SNP selection and adjustment for population structure in high dimensional prediction models by Sahir Rai Bhatnagar, Yi Yang, Tianyuan Lu, Erwin Schurr, JC Loredo-Osti, Marie Forest, Karim Oualkacha and Celia MT Greenwood.

We thank the reviewers for their constructive comments which we believe has significantly improved our manuscript. In this document, we reproduce the reviewers comments in italic font, and provide our response to each of them below. We have also highlighted our changes in the manuscript with bold text.

1 Reviewer 1

The authors demonstrate a method for genome-wide association that combines mixed-model based control of population structure or genetic relatedness with multi-variate regressions. The idea is to increase power for GWAS applications by including multiple markers at once while accounting for structure. Previous tools for this application are somewhat limited, so this method aims to provide a more comprehensive and computationally efficient tools. They include a well-structured R package to implement their method. This is an important topic, and their software is much faster and easier to use than existing tools. However I have several comments on their presentation, particularly the comparisons with existing methods and some limitations of the simulations:

1.1 Major issues

1. *I think that the novelty of the method is not made clear in the paper. The model itself appears virtually identical to that of the glmmlasso R package (Schelldorfer et al 2011) except limited to one random effect – the objective function appears identical, and those authors also derived a block coordinate gradient descent algorithm, though I don't know if they are identical. The addition here seems mostly in terms of computational efficiency and flexibility (combinations of L1 and L2 penalties vs just L1 for Schellendorfer). Using the SVD to rotate and diagonalize the LMM so that the random effect covariance matrix is diagonal is a very useful addition and makes the software very fast, but this is not really emphasized in the methods.*

   We agree with the reviewer that the model is almost identical to that of the glmmlasso, but limited to one random effect. The main contribution here is rotating the response vector Y and the design matrix X by the eigen vectors of the kinship matrix, which results in a diagonal covariance matrix. As we mentioned in the discussion, from a

practical point of view, there is currently no implementation that provides a principled way of determining the sequence of tuning parameters to fit, nor a procedure that automatically selects the optimal value of the tuning parameter. We have added this part to the methods:

> We note here that the main difference between the proposed model, and the `lmmlasso` (Schelldorfer et al. 2011), is that we are limiting ourselves to a single unpenalized random effect. Another key difference is that we rotate the response vector Y and the design matrix X by the eigen vectors of the kinship matrix, resulting in a diagonal covariance matrix which greatly speeds up the computation.

2. *Also, the BSLMM model (Zhou et al 2013 Plos Genetics) is also effectively a variable selection LMM, though it's Bayesian instead of frequentist and requires MCMC so is slower. But it's performance could be compared to ggmix and the two-step LASSO presented in this paper.*

   Thank you for pointing us to the `BSLMM` model. We have added this model to our comparisons for both the UK Biobank example and the GAW20 example. We were not able to consider it in our simulations because the GEMMA package requires BIMBAM or PLINK format, and all of our simulation study was conducted and designed in `R`. We also were not able to apply `BSLMM` to the mouse crosses example for the same reasons. From our understanding, `BSLMM` is focused more on phenotype prediction than variable selection. This was confirmed by our real data analyses, where a substantially large number of SNPs had non-zero effect sizes. We have added the following to the main text:

   > We additionally applied a Bayesian Sparse Linear Mixed Model (`BSLMM`) implemented in the GEMMA package to derive a polygenic risk score on the training set. Subsequently, we found that although the `BSLMM`-based polygenic risk score leveraged the most SNPs, it did not achieve a comparable prediction accuracy as the other three methods (Figure 3B).
   >
   > Again, we applied the `BSLMM` method by iteratively preforming five-fold cross-validation on each of the 200 simulated replicates. We found that the `BSLMM` achieved a lower cross-validation RMSE compared to the other methods (Table 2). However, this relatively higher prediction accuracy relied on approximately 80% of the 51,104 SNPs supplied given the nature of this method. This may suggest overfitting in this dataset. It is also noteworthy that we did not adjust for age and sex in the `BSLMM` modeling, as the current implementation of the method in the GEMMA package does not allow adjustment for covariates.
   >
   > We did not apply the `BSLMM` method to the mouse crosses data because the microsatellite marker-based genotypes could not be converted to a BIMBAM or PLINK format that the package demands.

3. *While this method is much faster than glmmlasso (and presumably BSLMM), how does it scale to large numbers of markers? Typical genomics datasets today contain >1e5-1e6 markers. The*

datasets used here seem to contain 10K-50K. There is some discussion about limitations of scaling to large N, but not large p.

Based on our recent experience, the current bottleneck for our method is large N due to the singular value decomposition. We expect a large p to also be an issue for $p > 1e6$. However due to the coordinate wise optimization, this is still less of an issue than the sample size. We have had some experience with the `biglasso` package which uses memory mapping strategies. Memory mapping is a strategy we are currently exploring for `ggmix`. We have added this to the discussion:

> There is also the issue of how our model scales with an increasing number of covariates (p). Due to the coordinate-wise optimization procedure, we expect this to be less of an issue, but still prohibitive for $p > 1e5$. The `biglasso` package (Zeng et al. 2017) uses memory mapping strategies for large p, and this is something we are exploring for `ggmix`.

4. *The first set of simulations include population structure and admixture. But all 10K markers are simulated in linkage equilibrium. This is an ideal situation for LASSO. But in real data, nearby markers will be partially correlated. How well does this method select the correct marker when there are other correlated markers? What does it do when the true causal variant is not in the data, but imperfectly correlated markers are (the typical GWAS setting)? Does it tend to select the nearest marker, or does it select a set of nearby markers? I can't tell in the GAW20 and mouse datasets the extent of the LD among markers and whether this is addressed there.*

   We think this is a very good point. We illustrate the LD structure among the markers in the GAW20 dataset and the mouse dataset separately in the following plots.

   On the left panel, we show the pairwise r^2 for 655 SNPs within a 1Mb-window around the causal SNP rs9661059 (indicated) that we focused on. The dotplot above the heatmap denotes r^2 between each SNP and the causal SNP. It is clear that although strong correlation does exist between some SNPs, none of these nearby SNPs is correlated with the causal SNP. The only dot denoting an $r^2 = 1$ represents the causal SNP itself. Therefore, it is reasonable that the models only picked up the true positive in most GAW20 simulations. We could not visualize all 51,104 SNPs because the LD matrix would then be huge, but we posit it is unlikely that a far-away SNP can be strongly associated with the causal SNP.

   On the other hand, we also show the pairwise r^2 for all microsatellite markers on the right panel. It is clear that many markers are considerably strongly correlated with each other, as we expected. However, in 200 bootstraps, we still observed that the two true positive loci (indicated) were the most often selected while none of the nearby markers were picked up in more than 50% of the bootstrap replicates. This shows that our method does recognize the true positives.

   In our newly added UK Biobank section, the markers we used are theoretically independent since Yengo et al. (2018) performed a COJO analysis which should have tuned down signals due to LD.

   We have added the following text to the discussion section, and have provided the LD plots in the supplement:

This particular example had many markers that were strongly correlated with each other. Nevertheless, we observed that the two true positive loci were the most often selected while none of the nearby markers were picked up in more than 50% of the 200 bootstrap replicates. This shows that our method does recognize the true positives in the presence of highly correlated markers. Nevertheless, we think the issue of variable selection for correlated SNPs warrants further study. The recently proposed Precision Lasso (Wang et al. 2018) seeks to address this problem in the high-dimensional fixed effects model.

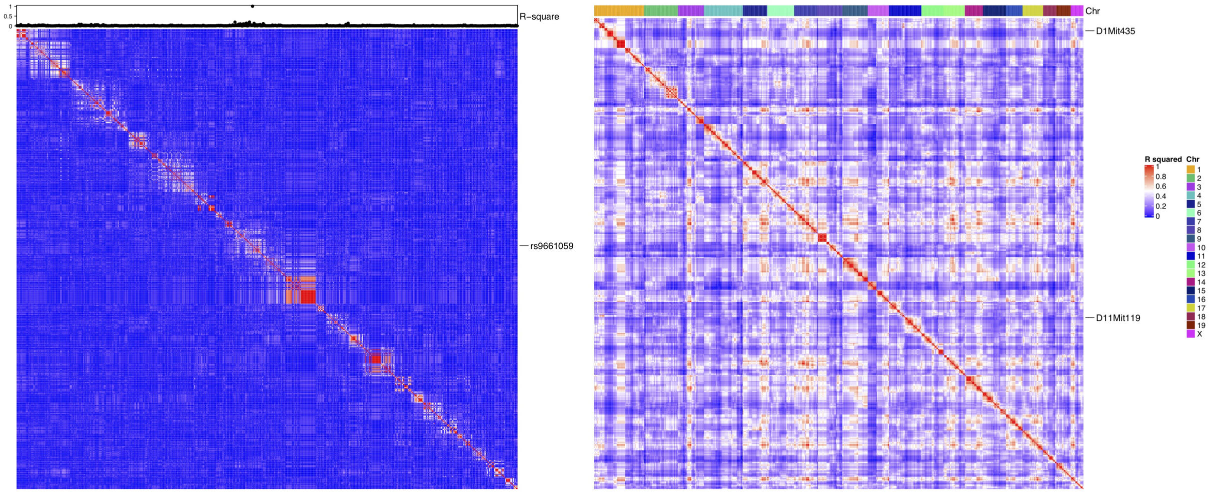

5. *Also, the simulation result that including the causal markers in the kinship matrix has little impact is encouraging. But the Yang et al 2014 paper, which discusses the impact of including causal markers in the estimated kinship matrix, suggests that this is only likely when N/M is large (ie as many or more individuals as markers). In the simulations presented here, N/M is <0.1, which is probably small enough that proximal contamination is not an issue. If N/M were 1, then the impact might be greater. Note: M is the effective number of markers after accounting for LD among markers.*

   We agree this is an important point which will require further study. It should be noted that the results in Yang et al. (2014) are focused on single locus association testing, and not the multivariable models being fit in this paper. It is not immediately clear to us, if and how, those results may be generalized to the multivariable setting.

   As was mentioned in our manuscript, a limitation of our approach is that it currently does not scale to large sample sizes, which makes it very computationally expensive to conduct simulations where the ratio of N/M is close to 1, particularly because the number of markers used to estimate the kinship matrix is often well beyond 10000. Our simulation scenarios with N/M = 0.1, were consistent with our real data analyses in GAW20 and UK Biobank. We have added the following text as a point of discussion:

   > As was brought up by a reviewer, the simulations and real data analyses presented here contained many more markers used to estimate the kinship

> than the sample size ($n/k \leq 0.1$). In the single locus association test, Yang el al. (2014) found that proximal contamination was an issue when $n/k \approx 1$. We believe further theoretical study is needed to see if these results can be generalized to the multivariable models being fit here. Once the computational limitations of sample size mentioned above have been addressed, these theoretical results can be supported by simulation studies.

1.2 Minor issues

1. *97: Wang et al 2011 does not treat the variance components as fixed, but iteratively estimates them along with beta.*

   Thank you, We have fixed the text to say:

   > However, methods such as the LMM-lasso are normally performed in two steps.

2. 142: How much of the total variance is accounted for by causal SNPs vs the background in the simulations?

   In our simulations, about 80% percent of the total variance was accounted for by the causal SNPs.

3. *355: $X_1, \ldots, X_N$ should each be transposed*

   Thank you. We have fixed this.

4. *Fig 5: I don't think that the red line is a p-value threshold. Maybe "significance threshold"? How can this be <100 if there were 200 bootstraps and significance required >50% inclusion?*

   We agree with the reviewer. The red lines denote significance thresholds. We have fixed this in the manuscript.

5. *I believe Eq (30) is wrong. The term V^{-1} shouldn't be present in the likelihood if b is included.*

   Thank you for this. We have fixed this in our manuscript as well as in our `R` package.

2 Reviewer 2

I have a few comments and suggestions:

1. *Page 2, Line 33: It this true: better sensitivity and specificity? What are you using to define sensitivity?*

   We agree with the reviewer that this part isn't clear. Related to comment #3, we have changed the simulation results to include the true positive rate at a fixed false positive rate of 5%. We have also change the text in the abstract as follows:

We develop a blockwise coordinate descent algorithm with automatic tuning parameter selection which is highly scalable, computationally efficient and has theoretical guarantees of convergence. Through simulations and three real data examples, we show that `ggmix` leads to more parsimonious models compared to the two-stage approach or principal component adjustment with better prediction accuracy. Our method performs well even in the presence of highly correlated markers, and when the causal SNPs are included in the kinship matrix. `ggmix` can be used to construct polygenic risk scores and select instrumental variables in Mendelian randomization studies.

2. *Page 7, line 143-163. I found the definitions of the different X matrices confusing. I think you can simplify to indexes: eg: causal for list of causal snp indexes, kinship for list of snp index in the kinship matrix, etc. I'd also refer the the snps as covariates and not label them as fixed, and state when the causal set is in the kinship set.*

   We agree with the reviewer that this notation is simpler. We have modified the main text as follows:

   For other parameters in our simulation study, we defined the following quantities:

   - n: sample size
   - c: percentage of causal SNPs
   - $\boldsymbol{\beta}$: true effect size vector of length p
   - $S_0 = \left\{j; (\boldsymbol{\beta})_j \neq 0\right\}$ the index of the true active set with cardinality $|S_0| = c \times p$
   - *causal*: the list of causal SNP indices
   - *kinship*: the list of SNP indices for the kinship matrix
   - $\mathbf{X}$: $n \times p$ matrix of SNPs that were included as covariates in the model

   We simulated data from the model

$$\mathbf{Y} = \mathbf{X}\boldsymbol{\beta} + \mathbf{P} + \boldsymbol{\varepsilon} \tag{1}$$

   where $\mathbf{P} \sim \mathcal{N}(0, \eta\sigma^2\boldsymbol{\Phi})$ is the polygenic effect and $\boldsymbol{\varepsilon} \sim \mathcal{N}(0, (1-\eta)\sigma^2\mathbf{I})$ is the error term. Here, $\boldsymbol{\Phi}_{n\times n}$ is the covariance matrix based on the *kinship* SNPs from n individuals, $\mathbf{I}_{n\times n}$ is the identity matrix and parameters σ^2 and $\eta \in [0,1]$ determine how the variance is divided between $\mathbf{P}$ and $\boldsymbol{\varepsilon}$. The values of the parameters that we used were as follows: narrow sense heritability $\eta = \{0.1, 0.3\}$, number of covariates $p = 5,000$, number of *kinship* SNPs $k = 10,000$, percentage of *causal* SNPs $c = \{0\%, 1\%\}$ and $\sigma^2 = 1$. In addition to these parameters, we also varied the amount of overlap between the *causal* list and the *kinship* list. We considered two main scenarios:

   (a) None of the *causal* SNPs are included in *kinship* set.
   (b) All of the *causal* SNPs are included in the *kinship* set.

3. *Page 7, line 159: Did you try a larger number of SNPs? In GWAS the number is much larger and PRS methods use much more than 50 independent SNPs for estimation. Page 10, line 183: if I understand your sparsity estimate correctly, with a causal rate of 1%, setting all B to zero would give a value of 99%?*

   We have added an entire section with a UK Biobank example. In this example, we derive a `ggmix` PRS for standing height, which is a highly polygenic trait, and compare it with the other methods. We found that an optimized `ggmix`-derived polygenic risk score that utilized the least number of SNPs was also able to better predict standing height with lower RMSE on the test set compared to `twostep`, `lasso`, and `BSLMM`.

   To the second point, yes that is correct. As was pointed out by other reviewers and your comment #4, this isn't a fair comparison. We have changed the simulation results to include the true positive rate at a fixed false positive rate of 5%, and have removed the correct sparsity estimate.

4. *Page 11, 188: I am curious why you only reported the 'optimal' value of the penalty parameter. Is your method outperforming in terms of sparsity because it just does a better job of selecting a sparse model? Your false positive rate is lower but the true positive rate is much lower. If one is searching for the set of true causal variants, they are usually willing to take the tradeoff of better sensitivity for weeding through the false positives. I would prefer to see curves of FP versus TP rates with the values at the optimal tuning parameter marked. In practice I found AIC/BIC somewhat conservative compared to CV or controlling for error rate via phenotype permutation.*

   Thank you for this comment. We reported both the optimal parameter and the one which gave a false positive rate of 5%.

   To the second point, we haven't explored CV for `ggmix`, particularly because our simulations and real data examples aren't pedigrees. Therefore, it is difficult to ensure that related individuals stay together in each fold. We think the general problem of cross-validation with correlated observations requires further study.

5. *Page 11, Line 196: I am sorry if I missed this somewhere, but how was the model tuned for the lasso and twostep in the training data? Did ggmix use the GIC in the materials and methods?*

   We agree with the reviewer that this was not clearly stated in the paper. We had only written that default settings were used for the lasso and twostep on page 6 line 139. The `glmnet` package that we used only has a cross-validation (CV) procedure. Therefore, this was the reason we used 10-fold CV in the training set for selecting the optimal tuning parameter for both the `lasso` and the `twostep` (note that the `twostep` also uses the `lasso` procedure). We have included the following text to clarify this:

   > We fitted the `lasso` using the default settings and `standardize=FALSE` in the `glmnet` package, with 10-fold cross-validation to select the optimal tuning parameter.

   We have also included this information in the caption of Table 1:

Model Size is the number of selected variables in the training set using the high-dimensional BIC for `ggmix` and 10-fold cross validation for `lasso` and `twostep`.

6. *Page 14, Line 243 typo- methods*

   Thank you. We have fixed this typo.

7. *Page 12: Figure 3. This data might be better summarized in a table that could include the additional data in the supplementary files.*

   We agree that the results in a single Table will be easier to parse compared to many figures. We have summarized the results in Table 1, which replaces Figure 3 as well as the Figures in the supplementary file.

8. *The math is a bit beyond my abilities, but I have previously read a paper that suggests maximizing the log likelihood, $-1/2[ln|V| + (Y - \boldsymbol{\beta}X)^\top V^{-1}(Y - \beta X)]$ subject to the L1/L2 norm penalty to control for relatedness in penalized regression methods for genetic data (where V is the variance covariance matrix). Is this essentially what you are doing?*

   Yes this is correct. The main advantage of our approach however, is that we are limiting ourselves to a single unpenalized random effect. Another key difference is that we rotate the response vector Y and the design matrix X by the eigen vectors of the kinship matrix, resulting in a diagonal covariance matrix which greatly speeds up the computation. We have added this clarification to our methods:

   > We note here that the main difference between the proposed model, and the `lmmlasso` (Schelldorfer et al. 2011), is that we are limiting ourselves to a single unpenalized random effect. Another key difference is that we rotate the response vector Y and the design matrix X by the eigen vectors of the kinship matrix, resulting in a diagonal covariance matrix which greatly speeds up the computation.

9. *It would have been interesting to use the set of related individuals in the UK Biobank on a few traits where PRS works well.*

   We agree with the reviewers that this is an interesting scenario. We have applied our algorithm to these related individuals in the UK Biobank and have summarised our results in a new section in the main text.

3 Reviewer 3

The authors present a penalized multi-variate regression model, ggmix, that jointly models multiple genotypes in mixed-model setup that incorporates the kinship or the genetic relationship matrix (GRM). It is an important problem in statistical genetics and I appreciate that the authors developed a comprehensive algorithm that simultaneously incorporates

population structure and variable selection problem. The methods are well described and the paper was easy to follow, but I have some concerns in the experiments and evaluation metric. Overall, I think the paper presents an important problem, but the results are not convincing.

1. *First, in simulation results, the 'correct sparsity' measure is basically 'accuracy' measure in binary classification problem of whether the regression coefficients are zero or non-zero. This 'accuracy' measure is often misleading when class distribution is imbalanced. For example, if 99% of coefficients are zero, you can get 99% accuracy by just classifying everything to be zero, which is clearly not a good model. I suggest adapting different measure, such as MCC. I can see that twostep and LASSO both maintains FPR at 0.05, which is why 'correct sparsity' is around 0.95. At the same time, we can see that LASSO and twostep achieve higher TPR. Also from a slightly different point of view, in Figure 3(D), comparing TPR at different points on FPR is not a fair comparison. To compare different methods in the context of TPR and FPR, either AUC or TPR at the same rate of FPR should be considered.*

   Yes we agree with the reviewer that indeed, this can be viewed as a binary classification problem with imbalanced classes. We have removed correct sparsity as a measure of performance from the manuscript. As suggested by the reviewer, to compare the methods in the context of true positive rate (TPR), we selected the largest tuning parameter that would result in a false positive rate (FPR) closest to 5%, but not more. The TPR results have been summarized in the first row of Table 1 of the manuscript.

2. *Intro is slightly misleading, especially in lines 107-109, because I first thought that ggmix takes out causal (i.e. selected) variables out of the relationship matrix, then I later realized that loss of power due to causal SNPs included in the GRM still happens in ggmix, but you aim to minimize the loss by joint modeling – but it is not clear why this would be the case. Is there any theoretical or simulation-based evidence that joint modeling achieves higher power in such a case?*

   We would like to clarify that lines 107-109 are referring to two-stage procedures, where in the first stage, the goal is to remove the dependence between observations. This is achieved by calculating the variance components from a LMM with a single random effect. These LMMs use the estimated kinship matrix from the individuals' genotypes to account for the relatedness but assumes no SNP main effects (i.e. a null model). The residuals from this null model can be treated as independent observations because the relatedness has been effectively removed from the original response. In the second stage, any model for independent observations can be used. Lines 107-109 are stating that two-stage procedure can suffer from a loss of power if the causal variant has been used in the modeling of the kinship matrix, because its effect will have been removed in stage one.

   This loss of power is confirmed by our simulation results and real-data analyses. First, our simulation results show that `ggmix` performs well even when the causal variants are included in the GRM. This is not the case for the `twostep` procedure. In Table 1, we see the TPR at a FPR of 5%, drops, on average, from 0.84 (when causal SNPs are not in GRM) to 0.76 (when causal SNPs are in GRM) for the `twostep`. The `lasso` with a principal component (PC) adjustment can also be seen as a joint model, because the PCs are derived from the same SNPs used to calculate the GRM. In the UK Biobank example,

we see that ggmix achieves a lower test set prediction error with a more parsimonious model compared to twostep, lasso and BSLMM. Here we used all genotyped SNPs to estimate the kinship matrix, among which, 1233 SNPs were included as fixed effects in the model. In the GAW20 example, both ggmix and lasso have better test set prediction performance compared to twostep. In the mouse crosses example, ggmix had superior variable selection results compared to both the twostep and lasso.

We have added the following text to the simulation results:

> The results are summarized in Table 1. We see that ggmix outperformed the twostep in terms of TPR, and was comparable to the lasso. This was the case, regardless of true heritability and whether the causal SNPs were included in the calculation of the kinship matrix. For the twostep however, the TPR at a FPR of 5%, drops, on average, from 0.84 (when causal SNPs are not in the kinship) to 0.76 (when causal SNPs are in the kinship).

and the following text to the discussion to clarify this point:

> We compared our method to the twostage procedure, where in the first stage, the dependence between observations is adjusted for in a LMM with a single random effect and no covariates (i.e. null model). The residuals from this null model can then be used in any model for independent observations because the relatedness has been effectively removed from the original response. We also compared our method to the lasso and BSLMM which are closely related to ggmix since they also jointly model the relatedness and SNPs in a single step. The key differences are that the lasso uses a principal component adjustment and BSLMM is a Bayesian method focused on phenotype prediction.

3. *In the mouse data, the model parameters were optimized so that the two loci are picked up, and then the evaluation metric is based on whether these two loci are picked up, which is circular.*

   We actually had two-fold assessment of model performance in this case. We first examined whether a model could pick up the two true positive using *any* λ - if it did not, we count as failure; if it did with some λ, we next examined which other loci were also picked up. The results summarized in the pie charts were based on the first criterion while the results illustrated in the plots of per-chromosome cumulative hits summarized extra information. This is now clarified in that section with the following text:

   > We then conceived a two-fold criteria to evaluate performance of each model. We first examined whether a model could pick up both true positive loci using some λ. If the model failed to pick up both loci simultaneously with any λ, we counted as modeling failure on the corresponding boostrap replicate; otherwise, we counted as modeling success and recorded which other loci were picked up given the largest λ. Consequently, similar to the strategy used in the GAW20 analysis, we optimized the models by tuning the penalty factor such that these two true positive loci were picked up, while the number of other active loci was minimized.

4. *In discussion, it was mentioned that leave-one-chromosome-out approach is possible, but has not been tried. What would be the compelling reason to model all chromosomes together in the proposed problem, especially when the model is still additive and trans-interaction term is not directly modeled?*

   For the LOO chromosome approach, the empirical kinship matrix would be different for each run, and therefore the regression coefficients would not be comparable. For this reason, it is not clear how we might generate a polygenic risk score from each of the different LOO models. It can also become computationally expensive to calculate and store 21 different kinship matrices. For the purposes of variable selection, we would also want to model all chromosomes together since the power to detect an association for a given SNP may be increased when other causal SNPs have been accounted for. Conversely, a stronger signal from a causal SNP may weaken false signals when modeled jointly, particularly when the markers are highly correlated as in the mouse crosses example. We have addded the following text to the discussion:

   > However, this approach is not possible if we want to model many SNPs (across many chromosomes) jointly to create, for example, a polygenic risk score. For the purposes of variable selection, we would also want to model all chromosomes together since the power to detect an association for a given SNP may be increased when other causal SNPs have been accounted for. Conversely, a stronger signal from a causal SNP may weaken false signals when modeled jointly, particularly when the markers are highly correlated as in the mouse crosses example.

Sincerely,

Sahir Bhatnagar

Sahir Rai Bhatnagar, PhD
Assistant Professor
Department of Epidemiology, Biostatistics and Occupational Health
Department of Diagnostic Radiology
McGill University
https://sahirbhatnagar.com
sahir.bhatnagar@mcgill.ca
514-398-2711
Purvis Hall, 1020 Pine Avenue West, H3A 1A2, Montreal, QC, Canada
(514) 398-2711 • sahir.bhatnagar@mcgill.ca • https://sahirbhatnagar.com/
