# Supplementary material for: Simultaneous SNP selection and adjustment for population structure in high dimensional prediction models
Source: PLoS Genet. 2020 May 4;16(5):e1008766. doi: 10.1371/journal.pgen.1008766 (PMC7224575; doi:10.1371/journal.pgen.1008766)
Supplement: Attachment — Submitted filename: ggmix_response_to_reviewers.pdf [file pgen.1008766.s007.pdf]

McGill
Faculty of Medicine
Department of Epidemiology, Biostatistics and Occupational Health
Department of Diagnostic Radiology

March 7, 2020

Re: Simultaneous SNP selection and adjustment for population structure in high dimensional prediction models by Sahir Rai Bhatnagar, Yi Yang, Tianyuan Lu, Erwin Schurr, JC Loredo-Osti, Marie Forest, Karim Oualkacha and Celia MT Greenwood.

We thank the reviewers for taking the time to carefully read our revised manuscript and provide additional comments. In this document, we reproduce the reviewers comments in italic font, and provide our response to each of them below. We have also highlighted our changes in the manuscript with bold text.

1 Reviewer 1

The authors have significantly revised their manuscript. I think that the inclusions of BSLMM in the comparisons is useful. However, I have a couple additional concerns.

1. *Are you sure you're extracting the model fit from BSLMM correctly? The specifics of use are not described in the methods. It's a Bayesian model, so gives a probability of inclusion of each SNP. From the prefix.param.txt file, you should use the gamma column to report the number of SNPs that cross a posterior inclusion probability threshold. If you count how many betas are != 0, that will likely be large. But this is not an accurate estimate of the number of markers included in the model. You can also get the posterior on the number of SNPs included from the prefix.gamma.txt file. If you're counting SNPs that are included, what posterior probability threshold are you using for inclusion? If you're reporting the % of SNPs included, are you reporting the posterior mean?*

   Thank you for this question. We have now clarified in line 305 that we used all SNPs with a positive posterior inclusion probability to predict the response, which is the default setting of GEMMA. We additionally tried imposing a stricter posterior inclusion probability threshold (0.05, 0.10 and 0.50) in order to improve feature selection. These thresholds however, resulted in overly sparse models as most SNPs had a low posterior probability. We have reported these additional results in Table 2 of the manuscript.

2. *Also, the authors didn't apply BSLMM to several of the analyses. There is a write.plink function in the snpStats package that could be used to write GEMMA-compatible input files from R. Also, the PhenotypeSimulator package seems to have a writeStandardOutput function that can write bimbam or Gemma output. Especially if the extraction of estimates of needs to be revised and in fact works more similarly to the other methods, then I would recommend applying it to all analyses (Biobank may be too large).*

Thank you for your suggestions. We realized that the data format can indeed be converted. We have removed that statement that such conversion was not possible. However, based on our understanding, BSLMM was not specifically developed for feature selection and we found the results were highly dependent on the choice of the arbitrary posterior probability threshold. We posit it would require extensive rationales and experiments to arrive at a reasonable threshold for each analysis, which is beyond the scope of our study. Therefore, we restricted such analyses to compare the prediction accuracy in the UK Biobank and GAW20 analyses.

3. *I am still a bit confused about when tuning parameters were set based on TPR / FPR and when based on GIC or CV or other direct methods. In real data, it's generally not possible to set based on TPR/FPR, but I think most of your comparisons now are done that way. I understand that the goal is to show that your model can work well, and so comparing to the truth is useful. But I think more clarity is needed about when you're demonstrating the true performance of the model vs when you're demonstrating how the model would actually be run by a practitioner who did not know any true positive effects going in.*

   Yes we absolutely agree with this comment. In the originally submitted version of our manuscript, all of our simulation studies were performed from a practitioner's point of view. As was pointed out by several reviewers, and in your comment, there are pros and cons to this approach. We have added the following text to the manuscript to clarify (Lines 185-189):

   > Note that in practice, this approach to selecting the tuning parameter is generally not possible since we do not know the underlying true model in advance. For real data, we suggest an information criterion approach described in Section 5.3.8 or a sample splitting approach such as the one we used for the UK Biobank analysis shown in Section 3.2.1.

4. *412-414. Is this statement true? Schelldorfer et al 2011 used what they called a "Block Coordinate Gradient Descent method" for their penalized LMM*

   To our understanding, we believe this statement to be true. We agree that Schelldorfer et al 2011 also developed a Block Coordinate Gradient Descent algorithm for solving their objective function. The key difference in our algorithm vs. theirs is the rotation of the response vector Y and the design matrix X by the eigen vectors of the kinship matrix, resulting in a diagonal covariance matrix. This leads to optimizing 2 variance parameters instead of q^* parameters as shown in Algorithm 1 of Schelldorfer et al 2011. It is for this reason we wrote:

   > ... in the specific context of fitting a penalized LMM for population structure correction with theoretical guarantees of convergence.

5. *501: I think that this is underselling your method. The main difference is that your method is orders of magnitude faster than lmmlasso, so it's actually usable. A secondary difference is that it is limited to only one random effect.*

   Thank you for highlighting this. We have changed the text as follows:

We note here that the main difference between the proposed model, and the `lmmlasso`, is that we rotate the response vector Y and the design matrix X by the eigen vectors of the kinship matrix. This results in a diagonal covariance matrix making our method orders of magnitude faster and usable for high-dimensional genetic data. A secondary difference is that we are limiting ourselves to a single unpenalized random effect.

6. *288: how do you get a p-value from ggmix?*

   We did not report any p-value based on ggmix. If it was the p-value in line 280 that you were referring to, that was a p-value obtained by running FaST-LMM to confirm previous findings in the literature.

2 Reviewer 3

The authors addressed most of my major concerns in this revision. I have one more comment:

1. *The authors first state they could not run BSLMM on simulated data and mouse data because of data format issues. Converting genotypes to PLINK format should be straightforward and should not be an issue. I understand for simulating large amount of data, it might be practically difficult, but I cannot understand why mouse microsatellite data cannot be converted to PLINK format. I do not think you need additional experiments with BSLMM but please remove the statement that converting the data to PLINK format is not possible.*

   Thank you for pointing this out. We had issues with the data format and now realized they can be converted to PLINK format. We have thus removed the statement. Meanwhile, as BSLMM was not specifically developed for feature selection, we did not apply it to the mouse microsatellite data analysis to avoid an overload of arbitrary criteria.

Sincerely,

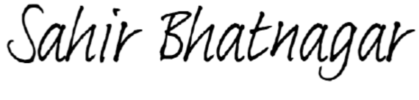

Sahir Rai Bhatnagar, PhD
Assistant Professor
Department of Epidemiology, Biostatistics and Occupational Health
Department of Diagnostic Radiology
McGill University
Purvis Hall, 1020 Pine Avenue West, H3A 1A2, Montreal, QC, Canada
(514) 398-2711 • sahir.bhatnagar@mcgill.ca • https://sahirbhatnagar.com/
